# Adaptive Emergence Patterns of Spot‐Tailed Earless Lizards: Influence of UV Light and Temperature on Diel Activity

**DOI:** 10.1002/ece3.71814

**Published:** 2025-07-17

**Authors:** E. Drake Rangel, Christin A. Moeller, Ruby A. Ayala, Scott E. Henke, David B. Wester, Cord B. Eversole

**Affiliations:** ^1^ Caesar Kleberg Wildlife Research Institute Texas A&M University‐Kingsville Kingsville Texas USA; ^2^ Department of Chemistry and Biology Texas A&M International University Laredo Texas USA; ^3^ Arthur Temple College of Forestry and Agriculture Stephen F. Austin State University Nacogdoches Texas USA

**Keywords:** emergence, *Holbrookia lacerata*, *Holbrookia subcaudalis*, Plateau spot‐tailed earless lizard, Tamaulipan spot‐tailed earless lizard, thermoregulation

## Abstract

We examined the emergence behavior of spot‐tailed earless lizards (STEL; *Holbrookia lacerata* and *H. subcaudalis*). Using controlled laboratory and seminatural experiments, we evaluated the effects of UV light, visible light, temperature, and prey activity on STEL emergence timing. Our results revealed that the combination of UV and visible light was the primary trigger for STEL emergence, aligning with peak light intensity and suggesting a physiological adaptation mediated through the parietal eye. In addition, the median time of emergence was 5 min after the initiation of LED and UV lights regardless of the time of day. Peak STEL activity occurred between 14:01 and 16:00 h with nearly 50% of STEL aboveground. This delayed emergence after sunrise allows for rapid thermoregulation, minimizing basking time while reducing predation risk by avoiding periods of peak predator (e.g., birds of prey, diurnal snakes) activity, which typically occurs during early morning and late afternoon hours, which is characteristic of arid systems. Moreover, the timing likely optimizes vitamin D_3_ synthesis, crucial for metabolic health, and minimizes energy expenditure associated with prolonged thermoregulation. STEL's emergence patterns contrast with sympatric species, typically occurring during midday rather than early morning or late evening hours, suggesting a reliance on unique diel niches. Little is known about STEL's ecology, particularly regarding their diel niche and activity patterns, which likely play a crucial adaptive role in species survival and success. Our findings demonstrate the importance of habitat‐specific light regimes in shaping the behaviors of reptiles and provide a model for understanding adaptive strategies in light‐sensitive species. As habitat degradation and climate change alter light and thermal environments, these behaviors may be disrupted, emphasizing the need for conservation practices that preserve open, sunlit habitats. This study contributes to our understanding of the ecological adaptations of reptiles and informs conservation strategies for species in arid and semiarid ecosystems.

## Graphical abstract

Our results of spot‐tailed earless lizards (STEL) light‐dependent emergence behavior not only illustrate adaptation to specific environmental cues, but also offer insights into the resilience and adaptability of reptiles inhabiting arid and semiarid ecosystems. Specifically, our study demonstrates how STEL's reliance on UV light shapes their behavior and suggests that their diel niche specialization reduces predation risks and interspecific competition.

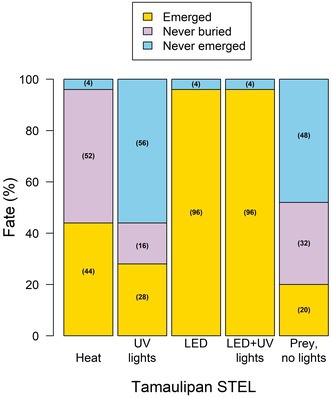

## 1 Introduction

The spot‐tailed earless lizards (STEL; *Holbrookia lacerata* [Plateau] and *H. subcaudalis* [Tamaulipan]) are diurnal, cryptic reptiles endemic to the arid and semiarid landscapes of west‐central and southern Texas (Axtell 1956). Historically widespread, these species have significantly declined in recent decades, thought to be largely due to habitat loss and degradation from agricultural expansion, land‐use changes, and habitat degradation resulting from brush encroachment and invasive vegetation (Rangel et al. 2024; Eversole et al. 2024). As a result, *H. lacerata* was recently considered for, although denied, U.S. federal protective listing, whereas *H. subcaudalis* remains under consideration for threatened status under the U.S. Endangered Species Act (Eversole et al. 2024; Rangel et al. 2024). Despite the conservation urgency, little is known about STEL's ecology, particularly regarding their diel niche and activity patterns, which likely play a crucial adaptive role in thermoregulation, predator avoidance, and overall species success (Hibbitts et al. 2021; Rangel et al. 2024). Although STEL are often described as burrowing lizards, they do not construct deep or permanent burrows. Instead, they typically bury themselves shallowly (e.g., within 1–3 cm of the soil surface) under loose substrate, allowing them to remain concealed while minimally shielded from environmental cues. This shallow burying behavior distinguishes them from true fossorial reptiles and suggests that full emergence is necessary for foraging, navigation, and predator avoidance. Given their active, visually oriented foraging strategy and cryptic coloration, timing full emergence to coincide with reduced predation risk may be as important as thermoregulatory cues. Furthermore, their limited anti‐predator defenses make them vulnerable to visually hunting predators. Thus, emergence behavior is likely more strongly shaped by predation risk and foraging requirements than by thermoregulatory needs alone. This knowledge gap complicates field surveys and population assessments, as current monitoring efforts often rely on generalized models of reptilian activity that may not capture the specific behaviors of STEL (Henke and Eversole 2021; Rangel et al. 2024). Addressing this gap is essential, as accurate ecological data can inform targeted management and conservation actions necessary to protect populations from further decline.

In reptiles, diel activity patterns are shaped by complex interactions of abiotic and biotic factors, such as temperature, light, humidity, and prey availability, which vary across seasonal and habitat gradients (Huey 1982). These patterns are particularly well‐documented in arid‐adapted lizards, which often employ bimodal or unimodal activity schedules that allow them to optimize activity above the soil surface while minimizing thermal stress (Pianka 1973; Sepúlveda et al. 2014). For instance, closely related species such as *Phrynosoma cornutum* and *Holbrookia maculata* typically avoid midday heat by concentrating activity in the morning and late afternoon hours, exhibiting predictable and adaptive patterns in response to intense environmental pressures (Fair and Henke 1998; Hager 2001). This behavioral pattern is consistent with the Metabolic Meltdown Hypothesis, which proposes that midday thermal extremes can exceed the physiological tolerances of ectotherms, compromising metabolic performance and survival (Huey and Kingsolver 2019). These responses are thought to balance the physiological demands of thermoregulation with behavioral adaptations to avoid predation, which can vary considerably across species, microhabitats, and climatic conditions (Herczeg et al. 2008; Weaver et al. 2024).

Past field observations suggest that STEL may diverge from this activity pattern, emerge later in the day, and remain active during peak ultra‐violet (UV) and ambient light intensity hours (Rangel, Henke, et al. 2022; Rangel, Moeller, et al. 2022). This distinct diel timing suggests an adaptive significance of these behaviors, although the ecological drivers that may influence STEL's emergence patterns are unstudied and unknown. Understanding STEL's specific diel activity and emergence patterns is crucial, not only for refining population monitoring techniques but also for expanding our knowledge of niche partitioning and behavioral adaptations in arid‐adapted herpetofauna. By identifying the environmental cues that trigger STEL emergence and when they are most active, we can apply basic ecological knowledge from experimental studies to survey methodology, timing, and field protocols to enhance detection rates and improve the accuracy and utility of survey results. Improved monitoring has direct conservation implications, as it allows for more accurate population assessments, which is an essential component for at‐risk species such as STEL (Urbano et al. 2024; Rangel, Henke, et al. 2022; Rangel, Moeller, et al. 2022). Moreover, as climate change continues to alter temperature and light regimes in arid landscapes, understanding STEL's diel niche may offer insights into how species reliant on specific microclimatic conditions are likely to respond to environmental shifts and how conservation and management strategies can be more precisely implemented.

To address these knowledge gaps, our goal was to experimentally determine the environmental factors that influence STEL emergence. Therefore, our objectives were to: (1) determine what environmental factors (i.e., temperature, UV light, ambient light, combination of UV and ambient light, or prey activity on surface) trigger STEL to emerge from burrows; (2) quantify the time elapsed from the initiation of the environmental factor of interest until STEL emergence, and (3) what time of the day STEL is most active and what diel time would be best for STEL surveys. Due to apparent delayed emergence by STEL in field situations, we hypothesized that (1) UV light is a primary trigger for STEL emergence from burrows, rather than visible light or temperature alone, because when the sun is lower in the sky (i.e., sunrise), more UV light is attenuated, and (2) emergence timing aligns with peak UV intensity as an adaptive strategy for thermoregulation and predator avoidance. Specifically, we hypothesize that UV intensity acts as a reliable cue for time of day, allowing them to synchronize above soil surface activity with periods when visual diurnal predators (e.g., birds of prey, diurnal snakes) are less active.

## 2 Methods

Through controlled laboratory experiments and seminatural trials, we manipulated light intensity, temperature, and prey availability to isolate their effects on STEL emergence behavior. By doing so, we aimed to clarify how diel cues influence activity in STEL, potentially offering a model for understanding adaptive emergence behaviors in other cryptic or understudied reptile species. To perform our experiments, we hand‐collected 45 spot‐tailed earless lizards (20 Plateau and 25 Tamaulipan STEL) from Tom Green (31.38194 N, −100.31361 W; WGS 84) and Nueces (27.71444 N, −97.84250 W; WGS 84) counties, respectively, via road‐cruising adjacent to habitat fragments near milo ( *Sorghum bicolor* ), maize ( *Zea mays* ) or cotton (*Gossypium* sp.) crop fields during May–June 2021. The roads consisted of either caliche or bare dirt between adjacent fields. STEL were transported to a research lab on the Texas A&M University–Kingsville campus, where STEL were housed in individual aquaria and behavioral studies were conducted.

### 2.1 Laboratory Setup and Conditions

Each of the 38‐L aquaria was filled with a 10 cm layer of sandy loam soil to simulate the natural substrate typical of STEL native habitat and for consistency across trials. The quantity of soil substrate was sufficient because STEL typically bury themselves just under the soil surface (Rangel, Henke, et al. 2022; Rangel, Moeller, et al. 2022). Temperature within each aquarium was maintained between 24°C and 35°C, reflecting the daytime temperature range in STEL's natural range (E. Rangel, unpublished data), using 100‐watt ceramic heat emitters (ZooMed Laboratories, San Luis Obispo, CA). The ceramic heat emitters provided a stable heat source without contributing additional light, allowing precise control of thermal conditions independently of light treatments. To simulate natural sunlight conditions, each aquarium was equipped with a ReptiSun 10.0 UVB, 13‐watt compact fluorescent lamp (ZooMed Laboratories), which provided ultraviolet light in the 290–320 nm range. LED lamps (ZooMed Repti Basking 100‐watt spot LED) also were positioned over each aquarium to replicate the intensity of ambient daylight without contributing additional UV radiation. Together, the UV and LED light sources were calibrated to approximate the midday light conditions typical of STEL habitats. All bulbs were mounted in ZooMed mini‐dome lamp fixtures, fixed above the aquaria to ensure consistent light distribution, and connected to programmable timers to control the onset and duration of each light treatment. To minimize external light interference and maintain the integrity of each lighting condition, all windows in the laboratory were covered, and overhead laboratory lights remained off throughout the experimental period. Black poster boards were placed around each aquarium to prevent cross‐illumination from adjacent treatments, ensuring each STEL was only exposed to its assigned environmental conditions. Additionally, individual aquaria were separated by dividers to limit visual cues or potential behavioral influences from neighboring aquaria. Behavioral observations were recorded using Geeni Vivid Indoor Smart Wi‐Fi Security Cameras (Merkury Innovations, New York, NY) positioned centrally above each aquarium. These cameras provided continuous video footage of STEL activity, capturing full views of inside each aquarium and ensuring high‐resolution monitoring of emergence behavior. Recordings were archived and reviewed posttrial for data analysis, allowing precise scoring of emergence times and behavior categorization. Live house crickets ( *Acheta domesticus* ) were introduced daily to stimulate prey response in the relevant treatment conditions, and shallow dishes with water were provided ad libitum to ensure STEL hydration throughout the study. This controlled setup enabled us to systematically manipulate UV light, LED light, heat, and prey availability across different trials, providing insights into the environmental factors influencing STEL diel emergence patterns under conditions closely resembling their natural habitat. Although we suspect that modality shifts could occur throughout STEL's active season, this study was not designed to address this.

### 2.2 Trial 1—Emergence Behavior Factors

STEL were randomly divided among each of the five treatments (i.e., heat only, UV light only, LED light only, a combination of UV and LED light, and presence of prey (i.e., crickets) maintained in darkness) and the fully negative control (i.e., aquaria maintained in darkness at room temperature; no UV and LED lights, no heat lamp, no crickets), given 2 days to acclimate to the treatment, and then on the third day behavioral data was recorded. To serve as replications, STEL were randomly rotated through each treatment until every STEL received every treatment. All treatments were initiated at 07:30 h. Time required from the initiation of a treatment until emergence onto the soil surface was recorded and categorized into six‐time blocks (i.e., < 2 min elapsed, 3–60 min elapsed, 61–120 min elapsed, and 121–720 min elapsed, never emerged, or never burrowed). We terminated experiments at 720 min because it represented a 12‐h interval, which was considered the maximum time a STEL displayed above ground behavior in the wild. During the 12‐h experiment, food (i.e., crickets) were withheld from STEL, except for the “presence of prey” treatment, which received crickets at the initiation of the trial. STEL within the other treatments were fed crickets immediately at the end of each experiment. We hypothesized that an increase in UV light would be the most significant factor in triggering STEL emergence.

### 2.3 Trial 2—Emergence Timing

A second behavioral study was initiated to determine if the timing of emergence is influenced by delaying the onset of the combination of UV and LED light. Twenty‐four STEL (12 Plateau and 12 Tamaulipan STEL) were used in Trial 2. Aquaria were set up as described in Trial 1, except that each aquarium was set to have the combination of UV and LED lights turn on at either 08:00, 10:00, or 12:00 h.

Eight STELs were randomly placed in each of the three light‐initiation cycles (i.e., 08:00, 10:00, and 12:00 h). During Trial 2, an acclimation period was not provided because we wanted to determine STEL's initial response to the UV and visible light stimulus rather than a potential habituated response. STELs were randomly rotated through each treatment until every STEL received all three treatments. Time required from the initiation of a treatment until emergence onto the soil surface was recorded and categorized into three blocks (i.e., < 2 min from initiation, within 3–30 min of initiation, or within 31–60 min of initiation). Based upon our observations from Trial 1, we hypothesized that STEL emergence would be triggered in the < 2‐min time block for each treatment (i.e., 08:00, 10:00, and 12:00 h).

### 2.4 Trial 3—Natural Lights

To verify that STEL behavior was not altered by artificial lighting, we evenly divided 40 STEL into 4, 2.5‐m circular diameter × 1‐m tall plastic mesocosms with 30‐cm deep of sandy loam soil in the bottom. Mesocosms were maintained outdoors, protected from predators within the avian flight cage of the Duane Leach Research Aviary at the Caesar Kleberg Wildlife Research Park, and STEL were allowed to maintain normal daily activities based on solar radiation and daylength for Kingsville, Texas (27.53222 N, −97.89036 W; WGS 84) during June–July, 2021. Ambient temperature and UV index were recorded hourly between 08:00 and 20:00 h during the 60‐day trial. Lizards were housed in groups of 10 by species, so 2 mesocosms contained 10 *H. lacerata* each and 2 mesocosms contained 10 *H. subcaudalis* each. Lizards were fed a diet of crickets and water *ad libitum* (Durtsche et al. 1997). STEL were not marked for individual identification so not to affect their normal behavior. A Geeni Vivid Smart Wi‐Fi Security Camera (Merkury Innovations, New York, New York 10,006) was mounted directly above the center of each tub so lizard activity could be monitored and recorded 24‐h/day. Diel hours were divided into 6, 2‐h blocks and spanned from 08:00–10:00, 10:01–12:00, 12:01–14:00, 14:01–16:00, 16:01–18:00, and 18:01–20:00 h. Time block when STEL first emerged from their burrows each day was recorded. Each day for 60 days, the instantaneous number of STEL that were above ground was counted once per time block within each mesocosm. Random time within each time block and each day was selected to count the number of STEL so to reduce potential behavioral similarities between days (i.e., if STEL were “creatures of habit” and behaved similarly each day at approximately the same time). The mean number of STEL aboveground during each time block was recorded throughout each 24‐h period. We hypothesized that STEL emergence would begin during time block 10:01–12:00 h (i.e., several hours after sunrise due to UV light attenuation caused by angle of the sun) and peak during time block 12:01–14:00 h during the peak of UV light intensity.

### 2.5 Statistical Analysis

For Trial 1, we used a linear mixed model analysis with repeated measures, where the fixed effects were species and treatment, random effects were lizard identification and rotation, and the dependent variable was minutes elapsed from the initiation of the treatment to STEL emergence from burrows. Treatments were heat, UV light, LED light, combination of UV and LED light, and prey availability. Because STEL behavior appeared altered by captivity or potential unnatural diel cycles (i.e., not all STEL would burrow and some individuals did not emerge), a post hoc test, the Cochran–Mantel–Haenszel (C–M–H) chi‐square analysis, was conducted. The C–M–H test is a contingency table‐based analysis that tested the differences among the treatments in frequencies of the three qualitative fates: emerged, never emerged, or never buried. Therefore, the C–M–H test was used to achieve objective 1, whereas the linear mixed model analysis answered objective 2. However, the linear mixed model analysis was only used to analyze data from STEL that did emerge; hence, sample sizes varied among treatments. All tests were considered significant at *p* ≤ 0.05. Trends in data were considered when *p*‐values were between 0.05 < *x* ≤ 0.10. Trends in data can suggest biological significance (Tacha et al. 1982).

For Trial 2, we used the Cochran–Mantel–Haenszel (C–M–H) chi‐square test as a qualitative analysis to determine differences in the frequencies of Plateau and Tamaulipan STEL that emerged within the time blocks of < 2, 3–30, and 31–60 min after the initiation of the combination of LED and UV lights. For the quantitative analysis, we transformed the time for emergence to log_10_ of minutes to reduce the skewness. We then conducted a nonparametric permutational analysis of variance where the fixed effects were species and treatment, random effects were lizard species and rotation, and the dependent variable was minutes elapsed from the initiation of the treatment to STEL emergence from burrows.

For Trial 3, we used a linear mixed model repeated measures analysis with permutational analysis of variance due to nonnormal distributions. Fixed effect was species (i.e., either Plateau or Tamaulipan STEL), random effects were STEL replication (i.e., mesocosms) and randomly selected time within time blocks, repeated measures included time and day effects, and the dependent variable was the number of STEL within a mesocosm that was aboveground at a specific time. From our Trials 1 and 2, the number of STEL that did not bury under UV and LED lights was small, and it also was assumed to be consistent across all mesocosms; therefore, nonburying STEL were considered nominal. Significance was inferred at *p* < 0.05. All means are reported ±1 standard error.

## 3 Results

### 3.1 Trial 1: Environmental Cues for Emergence

**FIGURE 1:**
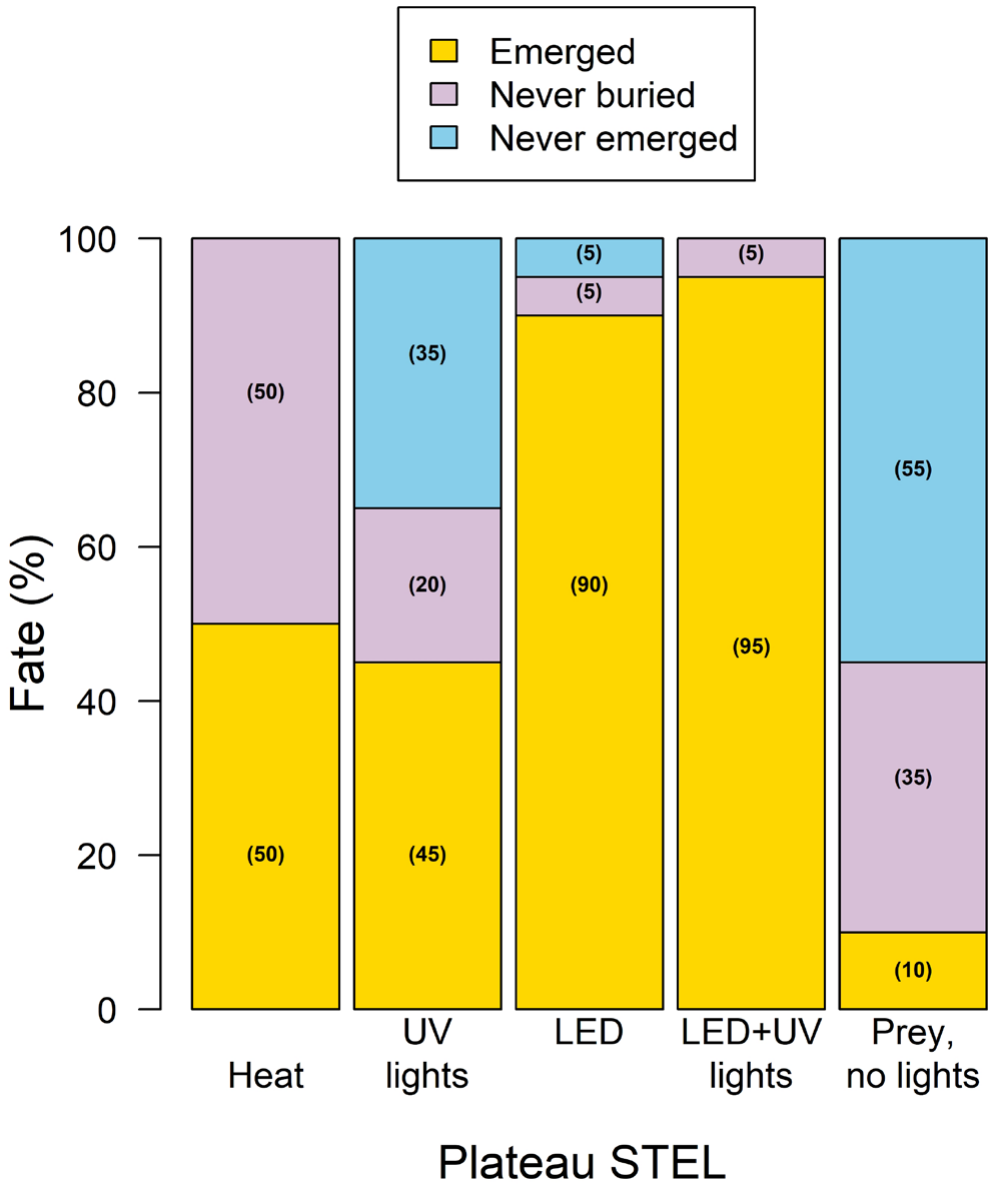
The outcome response of captive Plateau spot‐tailed earless lizards ( *Holbrookia lacerata* ) to illicit emergence from underground burrows when exposed to environmental factors of heat, ultra‐violet (UV) light, LED light, combination of UV and LED light, and prey.

**FIGURE 2:**
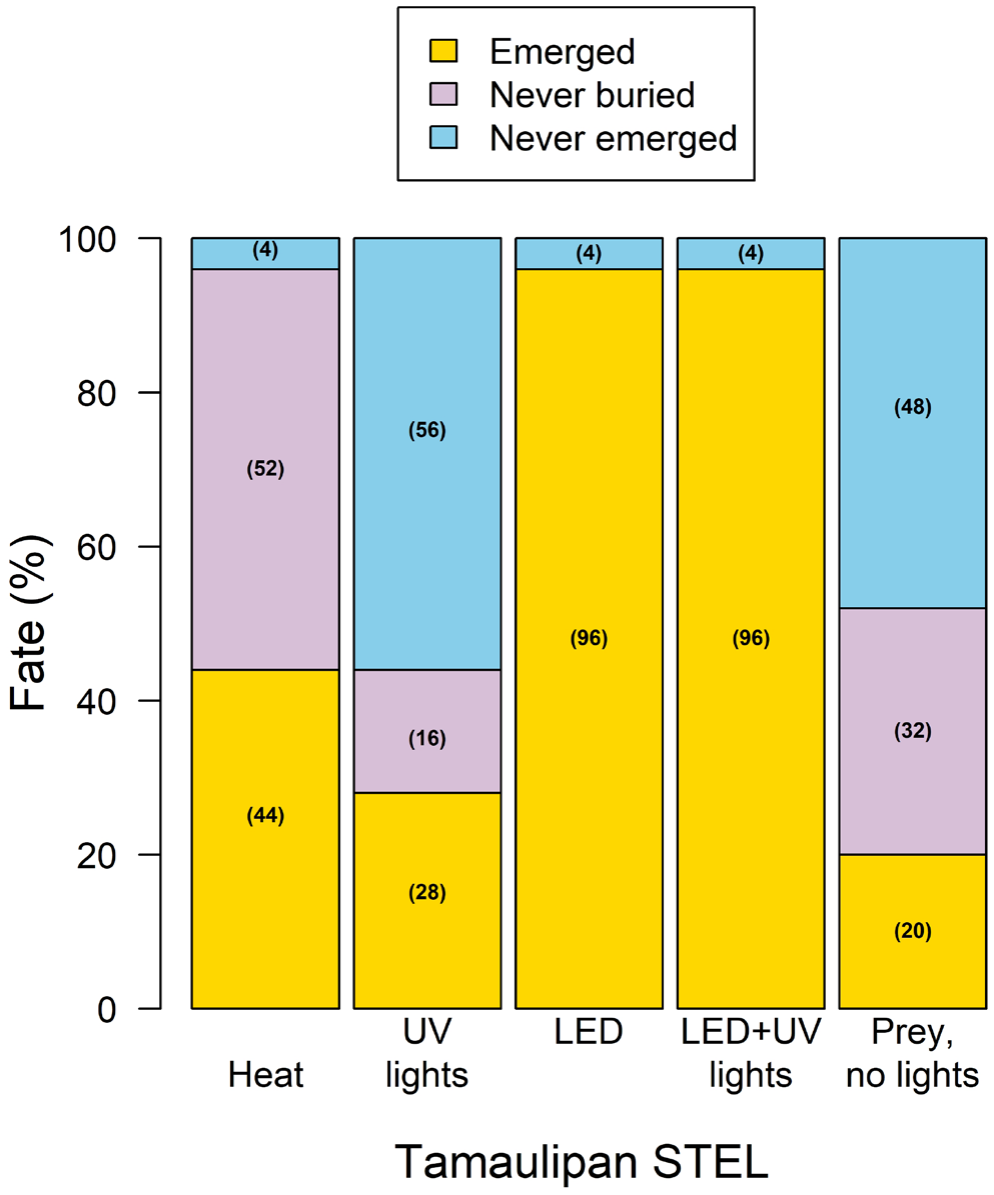
The outcome response of captive Tamaulipan spot‐tailed earless lizards (*Holbrookia subcaudalis*) to illicit emergence from underground burrows when exposed to environmental factors of heat, ultra‐violet (UV) light, LED light, combination of UV and LED light, and prey.

The Cochran–Mantel–Haenszel (C–M–H) chi‐square analysis revealed a marginal association between treatment type and emergence fate for Plateau STEL (*p* = 0.0645), indicating that different environmental cues may elicit varying emergence responses. For Plateau STEL, the combination of UV and LED light triggered the highest emergence rate, with 95% of individuals emerging, similar to LED light alone at 90% emergence (Figures 1 and 2). In contrast, lower emergence rates were observed with heat alone (50%), UV light only (45%), and prey movement (10%). For Tamaulipan STEL, however, no significant association was observed between treatment type and emergence fate (*p* = 0.76). Similar patterns were observed, with emergence rates of 95% and 90% under UV + LED light and LED light alone, respectively, while heat, UV light only, and prey movement led to 50%, 45%, and 20% emergence rates, respectively (Figures 1 and 2).

A linear mixed model analysis indicated no significant difference between species (*F*_1,8_ = 0.13, *p* = 0.73) or species × treatment interactions (*F*_4,28_ = 0.91, *p* = 0.47) for time to emergence across treatments, leading us to pool data from both species for further analysis (Table 1). A significant main effect of treatment on time to emergence was observed (*F*_4,28_ = 23.5, *p* < 0.0001). Pairwise comparisons showed that UV + LED light (12.0 ± 22.9 min) induced the quickest emergence time, significantly faster than all treatments except LED light alone (77.3 ± 23.3 min, *p* = 0.61 for Plateau STEL and *p* = 1.00 for Tamaulipan STEL). Emergence times were progressively longer for prey movement (95.0 ± 62.4 min), heat (113.0 ± 32.6 min), and UV light only (432.9 ± 37.6 min).

**TABLE 1:** Emergence behavior of spot‐tailed earless lizards (20 Plateau; *Holbrookia lacerata* and 25 Tamaulipan; *H. subcaudalis*) exposed to heat, ultra‐violet light, 18‐watt LED light, darkness with prey (i.e., crickets), and LED and ultra‐violet light combinations. Numbers represent the number of lizards (percent) that emerged immediately (within 2 min), 3–60, 61–120, and 121–720 min after the onset of an emergence factor.

Time	Heat	UV light	LED light	LED + UV light	Dark + prey
< 2 min	2 (4.4)	0 (0.0)	3 (6.7)	26 (57.8)	1 (2.2)
3–60 min	11 (24.4)	3 (6.6)	30 (66.7)	14 (31.1)	4 (8.9)
61–120 min	5 (11.1)	2 (4.4)	4 (8.9)	2 (4.4)	1 (2.2)
121–720 min	3 (6.6)	11 (24.4)	5 (11.1)	1 (2.2)	1 (2.2)
Never burrowed	23 (51.1)	8 (17.8)	1 (2.2)	1 (2.2)	15 (33.3)
Never emerge	1 (2.2)	21 (46.7)	2 (4.4)	1 (2.2)	23 (51.1)

### 3.2 Trial 2: Emergence Timing

**FIGURE 3:**
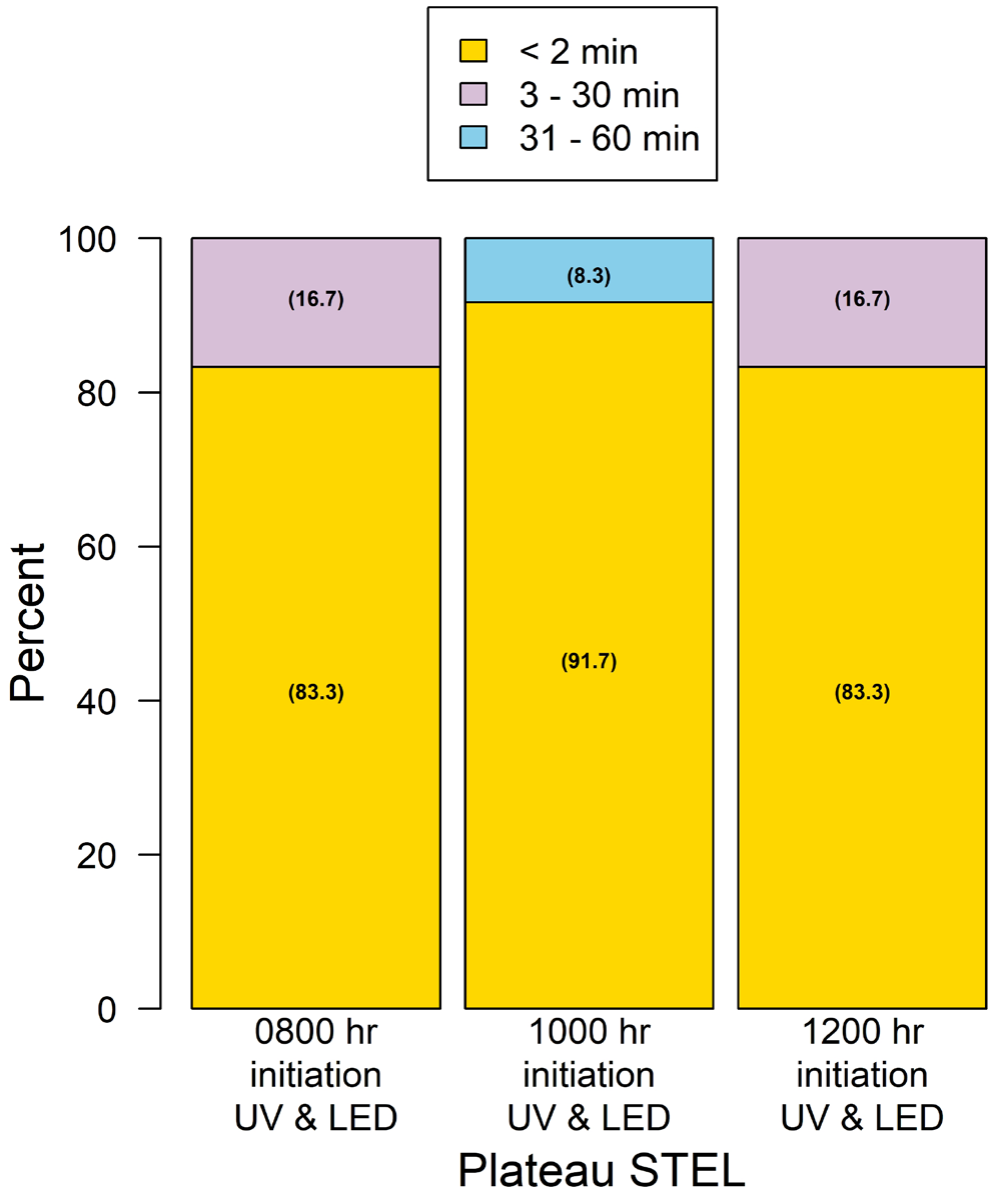
The outcome response of captive Plateau spot‐tailed earless lizards ( *Holbrookia lacerata* ) to illicit emergence from underground burrows when exposed to a combination of UV and LED light at three initiation times.

**FIGURE 4:**
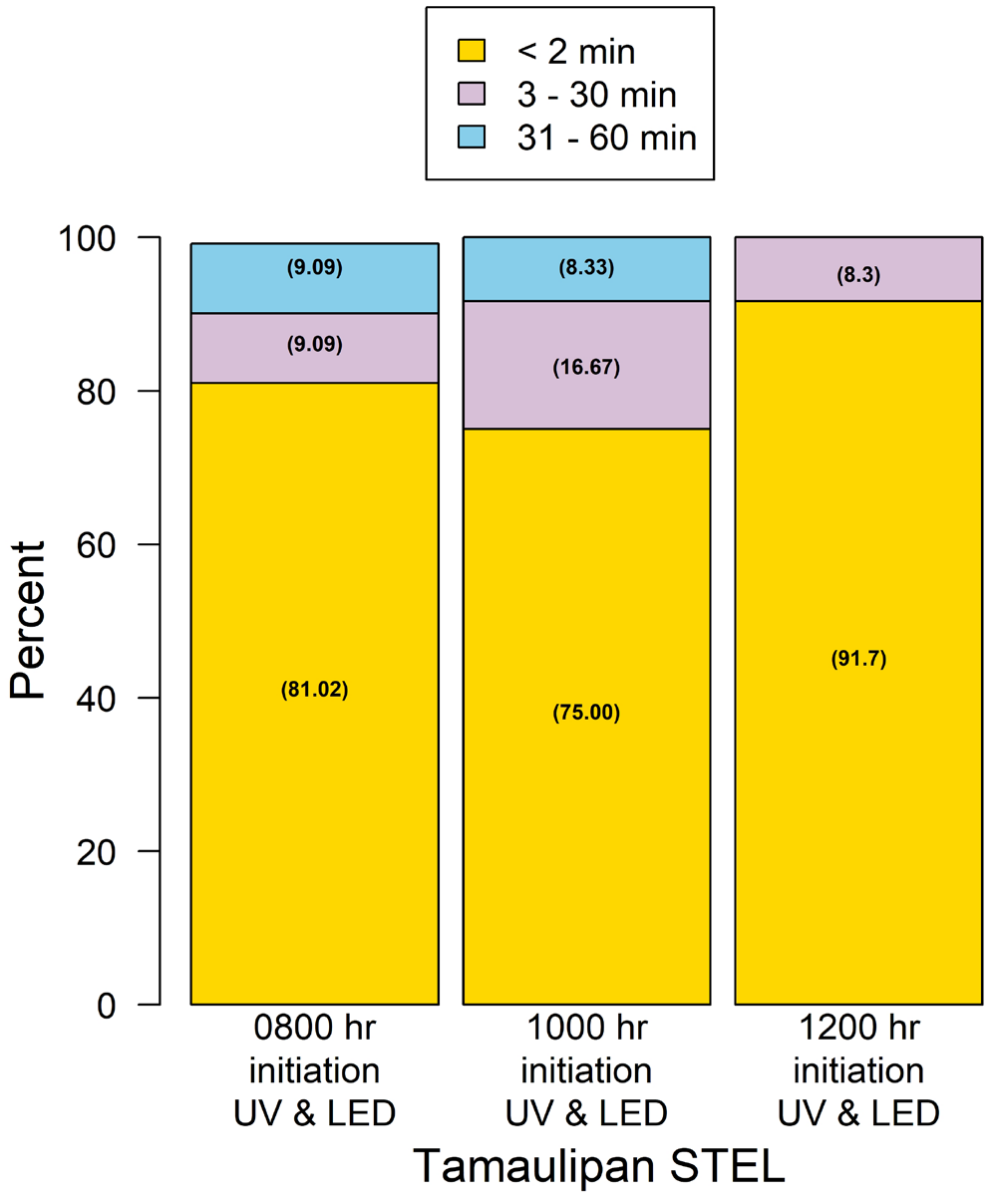
The outcome response of captive Tamaulipan spot‐tailed earless lizards (*Holbrookia subcaudalis*) to illicit emergence from underground burrows when exposed to a combination of UV and LED light at three initiation times.

In Trial 2, we examined whether the timing of light onset influenced STEL emergence (Table 2). Effects between species were not observed in emergence frequencies across treatments (Plateau STEL: *p* = 1.00; Tamaulipan STEL: *p* = 0.12). For Plateau STEL, the frequency of individuals emerging within < 2 min, 3–30 min, and 31–60 min was 83.3%, 16.7%, and 0% for LED and UV lights initiated at 08:00 and 12:00 h, and 91.7%, 0%, and 8.3% at 10:00 h (Figure 3). Similarly, for Tamaulipan STEL, emergence frequencies were 81.0%, 9.1%, and 9.1% at 08:00 h; 75.0%, 16.7%, and 8.3% at 10:00 h; and 91.7%, 8.3%, and 0% at 12:00 h (Figure 4).

**TABLE 2:** Number of spot‐tailed earless lizards (12 *Holbrookia lacerata* and 12 *Holbrookia subcaudalis*) (*N* = 24) and how quickly they emerged following initiation of UV and LED lights. Numbers represent the number of individual lizards that emerged in < 2, 3–30, and 31–60 min following illumination at 08:00, 10:00, and 12:00 h. Dashes represent times before the initiation of UV and LED lights.

	08:00 h			10:00 h			12:00 h		
Time (min)	0–2	3–30	31–60	0–2	3–30	31–60	0–2	3–30	31–60
08:00 h^a^	19	3	1	—	—	—	—	—	—
10:00 h	0	0	0	20	2	2	—	—	—
12:00 h	0	0	0	0	0	0	21	2	1

^a^ One spot‐tailed earless lizard never emerged above ground within 12 h after the initiation of UV and LED lights.

For the quantitative analysis, a significant main effect of treatment on time to emergence was observed (*F*_2,59_ = 10.6, *p* = 0.02). Across all treatments, the 0.75 quartile for emergence time was 2 min, indicating that 75% of individuals emerged within 2 min of light onset. Even at the 0.90 quartile, emergence times remained short, with median times of 5, 7, and 3 min after the initiation of LED and UV lights. STEL emerged rapidly following the onset of LED and UV lights, regardless of the time of day.

### 3.3 Trial 3: Natural Light Conditions

**FIGURE 5:**
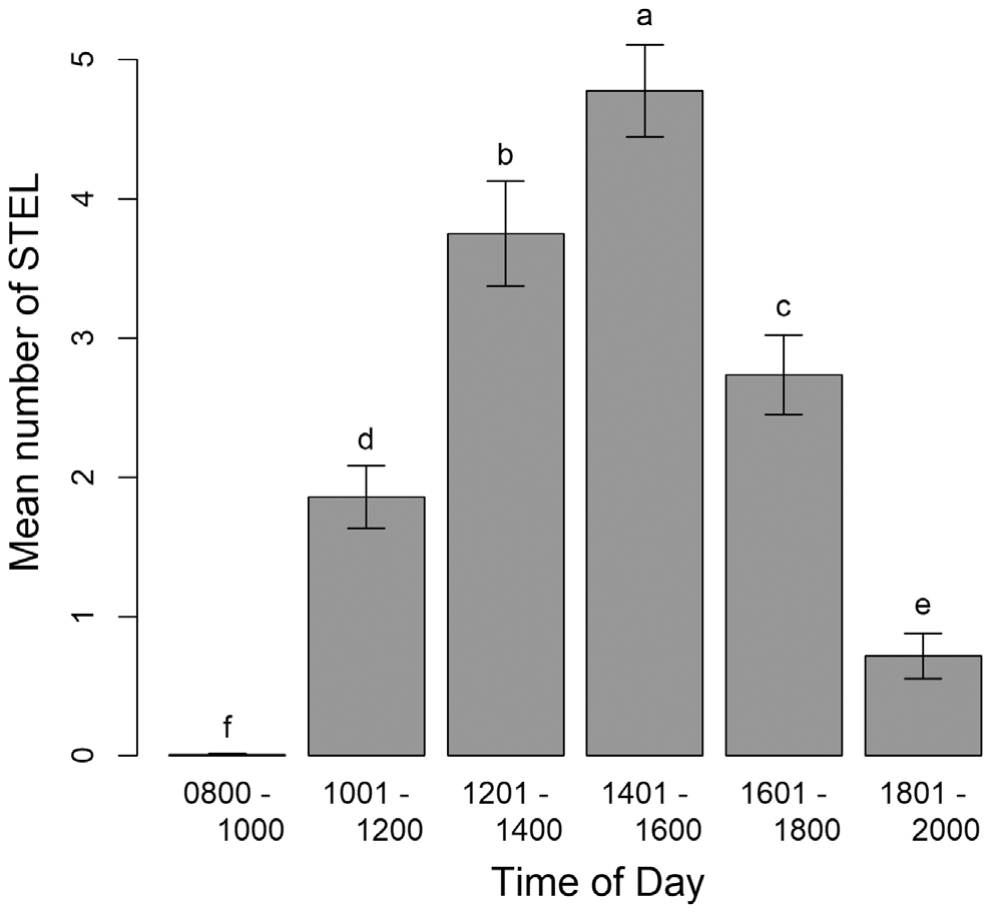
Mean number of spot‐tailed earless lizards ( *Holbrookia lacerata* (Plateau STEL) and *H. subcaudalis* (Tamaulipan STEL)) observed aboveground within the 4, 2.5‐m diameter mesocosms out of the known 10 STEL/mesocosm during June–July 2021.

Under natural light conditions, STEL exhibited a consistent diel pattern, with emergence not occurring until approximately 11:00 h (Range: 10:48–11:27 h) on any given day (Figure 5). The exception was one STEL that emerged at 09:56 out of the 40 STEL within the mesocosms during the 60‐day trial. Post‐emergence, individuals remained intermittently active, burying and reemerging throughout the day.

A linear mixed model analysis showed no main effect of species (*F*_1,2_ = 0.014, *p* = 0.99) or species × day × time interaction (*F*_145,300_ = 1.12, *p* = 0.20) on aboveground activity. However, significant effects of day (*F*_29,58_ = 8.60, *p* = 0.001), time (*F*_5,300_ = 1303.0, *p* = 0.001), and their interactions (day × time: *F*_145,300_ = 1.51, *p* = 0.004; day × species: *F*_29,58_ = 2.54, *p* = 0.005) were observed, indicating that diel activity patterns varied across days, likely due to weather conditions such as fluctuations in cloud cover.

**FIGURE 6:**
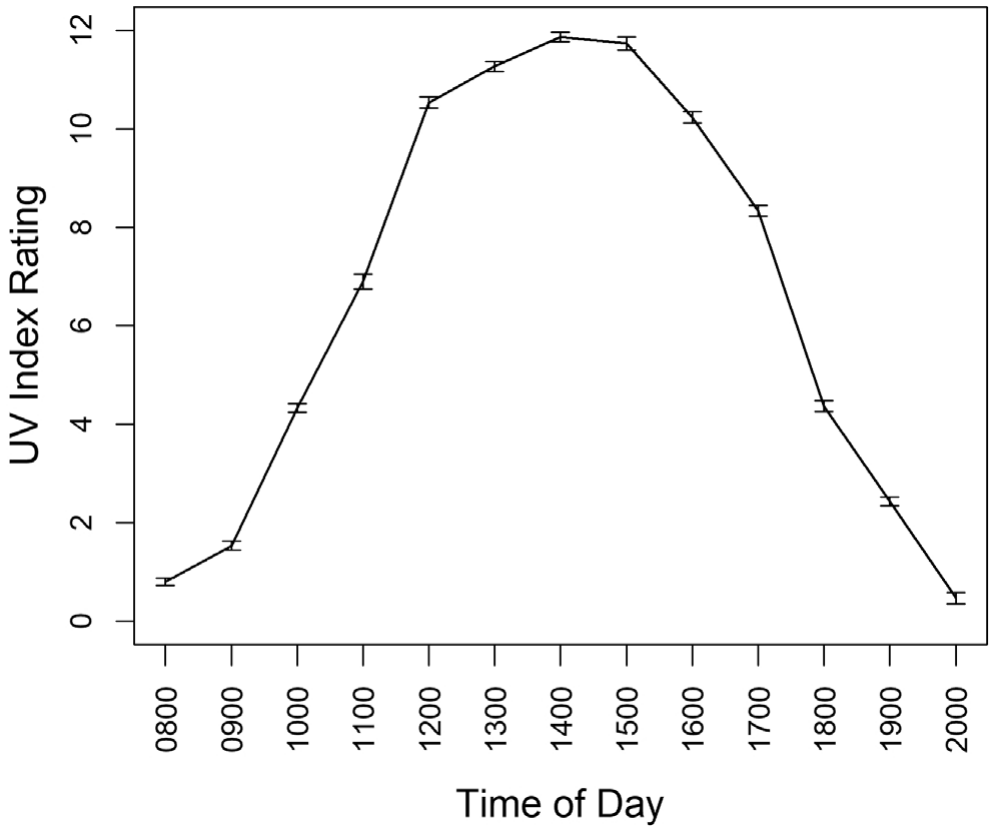
Mean hourly UV Index for Kingsville, Texas, during June–July 2021, obtained from the National Oceanic and Atmospheric Administration (https://www.noaa.gov/tools‐and‐resources/weather‐and‐climate‐resources/texas).

**FIGURE 7:**
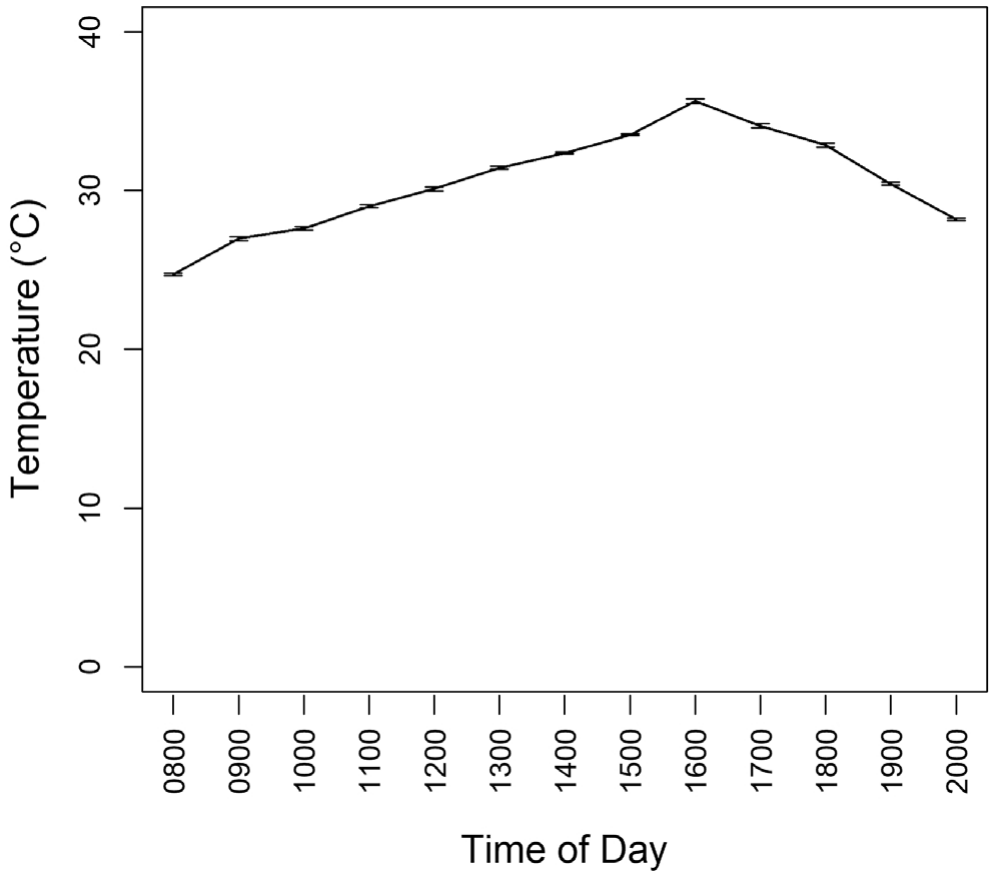
Mean hourly temperature (°C) for Kingsville, Texas, during June–July 2021, obtained from the National Oceanic and Atmospheric Administration (https://www.noaa.gov/tools‐and‐resources/weather‐and‐climate‐resources/texas).

Peak STEL activity occurred between 14:01–16:00 h, with an average of 4.75 ± 0.5 individuals aboveground, followed by 12:01–14:00 h (3.75 ± 0.5), 16:01–18:00 h (2.78 ± 0.5), 10:01–12:00 h (1.88 ± 0.4), 18:01–20:00 h (0.72 ± 0.3), and lastly 08:00–10:00 h (0.12 ± 0.02; Figure 5). STEL were inactive during nighttime and early morning hours (20:01–08:00 h), with a peak detection probability of ~48% between 14:01–16:00 h, making this the optimal survey window for field observations. The activity of STEL coincided with the UV Index. Mean UV Index was near 1.0 at 08:00 h, rose to 8.0 by 11:00 h, peaked above 12 between 13:00–15:00 h, and steadily dropped to near 0 by 20:00 h (Figure 6). Mean temperature was 25°C at 08:00 h, rose to about 35°C between 16:00–17:00 h, then decreased to 30°C by 20:00 h (Figure 7).

## 4 Discussion

Our study demonstrates that STEL emergence behavior is uniquely influenced by a combination of UV and visible light, resulting in a delayed diel activity pattern that differs from the bimodal activity seen in sympatric or closely related reptiles. Unlike related species that commonly emerge at dawn or exhibit two active periods, STEL appear to delay their surface emergence until the late morning or early afternoon, coinciding with peak UV and light intensity. This distinctive timing demonstrates an adaptation that enables STEL to rapidly thermoregulate by maximizing solar radiation absorption, rather than thermal heat emission from the surface, which allows them to quickly reach optimal body temperatures with minimal basking time. While our findings highlight the role of UV and visible light in triggering emergence, it is also possible that STEL passively thermoregulate while shallowly buried by absorbing heat radiating from the ground surface (i.e., conduction). When not pursued by predators, STEL bury themselves tail‐first by quickly shimmering their body until they are just under the soil surface (Rangel, Henke, et al. 2022; Rangel, Moeller, et al. 2022). This would reduce the need for prolonged surface basking and could explain their ability to emerge rapidly and become active with minimal delay. This efficiency likely reduces the duration of exposure on the surface; therefore, minimizing their vulnerability to predation. Additionally, by emerging later in the day, STEL may reduce encounters with primary predators, such as diurnal snakes and predatory birds, which are more active in the early morning and evening hours (i.e., sunrise and dusk; Craig 1978). The delayed emergence timing observed in the wild aligns closely with adaptive thermoregulation strategies seen in desert reptiles (Pianka 2017). For example, in arid environments, reptiles often face a narrow window for optimal basking, balancing the need to reach target body temperatures with the risks of thermal stress and predation (Pianka 2017). This diel activity shift suggests that STEL have evolved to balance thermoregulatory needs with predator avoidance, which optimizes their activity timing within the ecological constraints of the arid habitats that they occupy (Pianka 2017; Lapiedra et al. 2024). Interestingly, STEL appear to diverge from the predictions of the Metabolic Meltdown Hypothesis by concentrating their activity during midday hours. This deviation suggests that STEL may possess unique physiological or behavioral adaptations that mitigate the risks typically associated with extreme heat exposure (Huey and Kingsolver 2019). Thus, STEL's delayed emergence demonstrates a divergence from sympatric species but also suggests that STEL occupy a distinct diel niche that reduces competition and predation pressures, a behavior that can significantly enhance survival rates (Craig 1978). Our results elucidate these interactions in shaping STEL behavior and ecology and demonstrate the importance of considering species‐specific adaptations when implementing conservation strategies and monitoring programs. We acknowledge that we did not explicitly measure basking time per STEL; therefore, we cannot address the quantity of thermoregulation time required by them. However, our data do highlight that STEL emerge much later during the day than many reptiles, and that their emergence coincides with UV light reaching its peak for the day.

The reliance of STEL on the combination of UV and visible light for emergence likely reflects a specialized physiological adaptation, mediated through the parietal eye, which is connected to the pineal gland and characteristic of many diurnal lizard species (Wada et al. 2012; Pianka 2017). This organ plays a crucial role in regulating circadian and seasonal rhythms by detecting changes in light intensity and duration (Tosini 1997). Specifically in STEL, this organ may enable individuals to sense gradual changes in ambient light intensity throughout the day, allowing them to specifically alter their emergence timing to coincide with optimal UV and visible light conditions. This precise light sensitivity may be particularly beneficial in the arid habitat that STEL occupies, where peak UV exposure aligns with favorable temperatures for rapid warming, facilitating efficient and optimal thermoregulation. Although UV light alone produced one of the slowest emergence responses in our trials, the combination of UV and visible light consistently triggered the most rapid and complete emergence behavior. This pattern suggests that visible light is the dominant cue for STEL emergence, with UV radiation acting to modulate or enhance the response rather than serve as a primary trigger. Importantly, the UV‐only treatment was not sufficient to induce strong emergence behavior, which aligns with the idea that a critical threshold of total irradiance—especially in the visible spectrum—is necessary to initiate emergence. However, the enhanced response seen in the UV + LED treatment implies that UV may play a synergistic role, potentially mediated by physiological mechanisms such as parietal eye sensitivity or vitamin D_3_ synthesis needs.

In natural conditions, UV and visible light intensity typically covary due to their shared dependence on solar elevation, but UV experiences greater attenuation when the sun is low. Therefore, the timing of STEL emergence during peak midday hours may reflect sensitivity to this combined light profile, where UV acts as a reinforcing cue, ensuring emergence aligns with optimal thermal and metabolic windows. Future work manipulating UV and visible light intensity gradients independently would help further parse their respective contributions and potential physiological thresholds involved in emergence behavior.

Beyond thermoregulation, UV radiation directly influences several critical physiological processes in lizards (Gould et al. 2018). One of the most significant roles of UV exposure is in the synthesis of vitamin D_3_, which is essential for calcium absorption and bone health (Watson and Mitchell 2014). In lizards, insufficient UV exposure can lead to metabolic bone disease and impaired reproductive health (Watson and Mitchell 2014). STEL preference for emerging during peak UV hours likely supports optimal vitamin D_3_ synthesis, thereby promoting overall health and success (Karsten et al. 2009). Such physiological needs may explain STEL's delayed emergence, as timing their activity to periods of intense UV light could allow them to more quickly meet their physiological requirements, reducing the need for prolonged basking sessions and consequently minimizing exposure to predators.

The STEL behavior we observed in our experiments may appear to sharply contrast with their natural emergence pattern (i.e., Trial 3), where STEL typically delay activity until around 11:00 h. However, our results suggest that under natural conditions, STEL rely not only on the presence of visible and UV light, but also on a gradual increase in intensity, with STEL emergence coinciding with peak intensity. The rapid response by STEL in laboratory trials, where light intensity was introduced at full strength immediately, demonstrates the critical role of light intensity thresholds in STEL's behavioral cues. Under natural conditions, the angle of the sun at sunrise causes an attenuation of UV light striking the earth's surface (Diffey 1991). As the sun rises and becomes closely perpendicular to the Earth's surface, less UV attenuation occurs, which increases UV intensity (Diffey 1991; Blumthaler et al. 1993). It appears that STEL have likely adapted to the natural progression of the Sun's UV light in their arid habitats (Gamble et al. 2015).

Moreover, by emerging later in the day, STEL avoids competing for basking sites and resources with sympatric species that typically follow bimodal or early morning activity patterns, such as *Phrynosoma cornutum* and *Holbrookia maculata* (Fair and Henke 1998; Hager 2001). This temporal partitioning could help reduce interspecific competition for thermal resources, thus allowing STEL to occupy a distinct thermal niche, optimizing their ecological role within these shared habitats.

This behavior also has implications for STEL's metabolic efficiency. By timing emergence to coincide with peak solar radiation, STEL can quickly reach body temperatures that support active foraging and other energy‐intensive activities, potentially reducing the need for additional basking later in the day (Avery et al. 1982). This efficient use of solar energy is also metabolically beneficial by conserving energy that would otherwise be expended on prolonged thermoregulation efforts (Avery et al. 1982). The refinement of the observed light‐dependent thermoregulation behavior suggests an adaptive strategy that maximizes the metabolic benefits of UV and visible light exposure while minimizing the ecological costs associated with predation and competition. In addition, delayed emergence also may demonstrate an indirect metabolic advantage by reducing basking time and thus allow more time for foraging and mating, therefore increasing the overall fitness and success of individuals.

The delayed emergence strategy may be particularly advantageous for STEL, given their limited defensive mechanisms. Unlike some reptiles with physical deterrents, such as spiny scales or the ability to squirt blood (e.g., *Phrynosoma cornutum* ), STEL rely primarily on cryptic coloration and burrowing behavior for protection (Rangel et al. 2024). Their cryptic camouflage allows them to blend with their environment, but this passive defense is successful only if they remain relatively concealed. By emerging only during midday hours, STEL may minimize the likelihood of detection by visually oriented predators, utilizing the midday heat as a protective buffer when predator activity decreases. Additionally, STEL's tendency to burrow at night or during unfavorable conditions (i.e., high predator activity, low temperatures, etc.) may allow them to detect environmental changes while remaining concealed from just below the soil surface. While STEL may detect some degree of light or temperature change while shallowly buried, full emergence is required for effective visual foraging and situational awareness. As active, diurnal foragers with limited physical defenses, STEL rely on visual cues to locate prey and detect predators. Emergence during midday likely reflects an adaptive strategy to synchronize surface activity with conditions that minimize predator encounters and maximize foraging efficiency, rather than a need to thermoregulate from a cold, deeply buried position.

The unique diel activity pattern observed in STEL has broader ecological implications such as potential resilience and vulnerability of STEL to environmental changes. STEL's dependence on specific UV and visible light cues demonstrates behavioral patterns that are specifically adapted to local environmental conditions. This sensitivity to light intensity, along with the precise timing of emergence, suggests that even minor changes in habitat structure or light availability could significantly impact STEL's behavior and survival. For instance, habitat degradation resulting from increases in canopy cover, urbanization, or thermal environment could alter the intensity and amount of light reaching the ground (Dormann et al. 2020). Such shifts might disrupt the light cues essential to STEL's emergence, potentially leading to disparities between their behavior and the optimal conditions for thermoregulation and predator avoidance (Radovics et al. 2023). Alternatively, the observed diel cues could potentially become maladaptive for STEL as a result of warming trends via climate change, leading to physiological issues related to overheating or indirect effects on weather patterns, habitat, and prey base (Eversole et al. 2024).

Conservation strategies focused on preserving open, sunlit areas are therefore likely critical for maintaining the natural diel niche of STEL and other light‐sensitive species. Unfortunately, throughout much of the geographic range of STEL, increases in ground and canopy cover as a result of brush and invasive grass encroachment are pervasive problems that are the root of many conservation challenges in the region (Wied et al. 2020). It is possible that due to its effect on diel patterns of STEL, this could be driving the observed declines in these species. As such, our results demonstrate the importance of habitat management practices that retain open, sunlit spaces in ecosystems where UV‐sensitive species are present. As landscapes become increasingly altered, the maintenance of natural light regimes could be crucial for preserving species that depend on specific diel cues, such as STEL. For instance, the preservation of sparsely vegetated areas or the management of invasive plant species that alter ground cover could help mitigate habitat loss and support the persistence of species that rely on open habitats and sunlight (Eversole et al. 2024). For example, STEL's unique timing of emergence, synchronized with midday light intensity, reflects an evolved adaptation that balances thermal demands, predator avoidance, and resource partitioning. Such behaviors highlight the importance of maintaining intact ecosystems that allow for the full expression of these adaptive strategies. As climate change and habitat alteration continue to alter ecosystems, studying the interactions between behavior, physiology, and environmental factors will be crucial for predicting the impacts on biodiversity and developing effective management and conservation strategies (Madliger et al. 2018; Choi et al. 2019). Strategies that incorporate an understanding of species‐specific light requirements may therefore play a key role in sustaining biodiversity in changing ecosystems (Dawson et al. 2011; Butt and Gallagher 2018).

The observed diel niche specialization in STEL demonstrates the need to tailor conservation practices to species‐specific behaviors. Conservation plans that involve habitat modification or restoration should carefully consider STEL's reliance on open, sunlit areas, as these environments provide the essential light cues that drive their emergence and activity. Habitat management practices that preserve or create open spaces with minimal canopy cover can help maintain the conditions necessary for STEL's midday activity patterns. This consideration is especially important in areas undergoing rapid vegetation encroachment, agricultural expansion, or urbanization, which can alter light availability and fundamentally disrupt the species' behavioral ecology. By ensuring that STEL habitats include unshaded, sunlit areas, natural resource managers can target the species' natural diel niche and behaviors that will increase long‐term population viability. Our study also highlights the importance of management strategies that consider the broader implications of landscape changes on light‐sensitive species. For example, the encroachment of invasive plant species can increase shading and alter microhabitats, reducing the availability of open basking sites and changing the light profile of STEL habitats.

Our results also have significant practical implications for field research and conservation efforts aimed at accurately assessing and managing STEL populations. For example, traditional survey protocols, which typically focus on early morning or late afternoon sampling to coincide with the activity patterns of many diurnal reptiles, may overlook STEL's unique midday activity, resulting in underestimations of population density and status. Our results suggest that surveys targeting STEL should be conducted when peak UV and visible light intensity aligns with the species' preferred activity window, which in southern Texas occurred during 10:00 and 16:00h. Incorporating this timing into field methodologies could substantially improve detection rates, leading to more accurate population assessments and enhancing the ability to monitor STEL over time. Such improvements are crucial for informing conservation status evaluations and allocating resources effectively in habitats where STEL populations are thought to be declining.

## 5 Conclusions and Implications

Our results of STEL's light‐dependent emergence behavior not only illustrate adaptation to specific environmental cues but also offer insights into the resilience and adaptability of reptiles inhabiting arid and semiarid ecosystems. Specifically, our study demonstrates how STEL's reliance on UV light shapes their behavior and suggests that their diel niche specialization reduces predation risks and interspecific competition. By relating the ecological implications of these adaptations, we can better understand the complex relationships between species and their environments and better incorporate this into management and conservation actions.

By focusing on the ecological and physiological drivers of species‐specific behavior, conservationists and natural resource managers can design more effective strategies that align with the natural behaviors and needs of target species. This study highlights a novel diel activity strategy in STEL, which deviates from the bimodal or unimodal patterns observed in many sympatric species. Our results contribute to the broader understanding of how STEL may partition ecological niches and have adapted to the arid environments in which they occur. This study demonstrates the importance of how physiological and behavioral adaptations can be integrated into ecological monitoring frameworks to refine population assessments and inform conservation strategies for cryptic or declining species. For STEL, understanding the role of midday light cues can guide population monitoring and survey protocol as well as efforts to prevent habitat loss and degradation that would otherwise disrupt their activity cycles and thermoregulatory processes.

## Author Contributions

**E. Drake Rangel:** data curation (supporting), investigation (supporting), methodology (supporting), project administration (supporting), writing – original draft (supporting). **Christin A. Moeller:** data curation (supporting), methodology (supporting), project administration (supporting). **Ruby A. Ayala:** methodology (supporting). **Scott E. Henke:** conceptualization (lead), data curation (equal), formal analysis (equal), funding acquisition (equal), investigation (equal), methodology (equal), project administration (equal), resources (equal), supervision (equal), writing – original draft (equal), writing – review and editing (equal). **David B. Wester:** formal analysis (lead), validation (lead), writing – review and editing (equal). **Cord B. Eversole:** conceptualization (equal), funding acquisition (equal), investigation (equal), methodology (equal), resources (equal), writing – original draft (lead), writing – review and editing (equal).

## Conflicts of Interest

The authors declare no conflicts of interest.

## Data Availability

The data is publicly available on fig.share under the heading STEL Emergent Data. Also the corresponding author will make data available upon request. https://figshare.com/articles/dataset/STEL_Emergent_Data/28207523.
